# Mental health, functioning, and quality of life in employees who worked in the office vs. from home during the first wave of COVID-19 in Brazil

**DOI:** 10.47626/2237-6089-2022-0537

**Published:** 2024-05-24

**Authors:** Silvia Dubou Serafim, Jéferson Ferraz Goularte, Giovana Dalpiaz, Marco Antonio Caldieraro, Adriane Ribeiro Rosa

**Affiliations:** 1 Laboratório de Psiquiatria Molecular Hospital de Clínicas de Porto Alegre Porto Alegre RS Brazil Laboratório de Psiquiatria Molecular, Hospital de Clínicas de Porto Alegre (HCPA), Porto Alegre, RS, Brazil.; 2 Programa de Pós-Graduação em Psiquiatria e Ciências do Comportamento Universidade Federal do Rio Grande do Sul Porto Alegre RS Brazil Programa de Pós-Graduação em Psiquiatria e Ciências do Comportamento, Universidade Federal do Rio Grande do Sul (UFRGS), Porto Alegre, RS, Brazil.; 3 Departamento de Farmacologia UFRGS Porto Alegre RS Brazil Departamento de Farmacologia, UFRGS, Porto Alegre, RS, Brazil.; 4 Programa de Pós-Graduação em Farmacologia e Terapêutica UFRGS Porto Alegre RS Brazil Programa de Pós-Graduação em Farmacologia e Terapêutica, UFRGS, Porto Alegre, RS, Brazil.

**Keywords:** COVID-19, depression, anxiety, post-traumatic stress disorder, functioning, quality of life, work from home, work in office

## Abstract

**Objective:**

Coronavirus disease 2019 (COVID-19) was declared a global pandemic early in 2020, followed by a period during which governments imposed strict social distancing measures to slow transmission. However, most essential services remained open, and those working in offices faced a higher risk of infection compared to those working at home. We compare the occurrence and potential determinants of mental health outcomes, functioning, and quality of life (QoL) in a subset of a sample of Brazilian individuals who worked from home and a subset who worked in the office during the first wave of COVID-19.

**Methods:**

Data were collected during the first wave of COVID-19, using an online survey to assess sociodemographic and clinical variables, functioning with the Digital Functioning Assessment Short Test (D-FAST), QoL with the European Health Interview Surveys QoL instrument (EUROHIS-QOL), depression with the Patient-Reported Outcomes Measurement Information System (PROMIS) depression instrument, anxiety with the PROMIS anxiety instrument, and stress symptoms with the Impact of Event Scale-Revised (IES-R) in a large sample comprising individuals who worked in offices (n = 1685) or worked from home (n = 1,338).

**Results:**

Analysis revealed that depressive and post-traumatic stress symptoms were less prevalent in individuals who worked from home and showed that they had higher functioning and QoL than those working in the office. Individuals who worked in the office were younger, more likely to be female, had lower household income, had low educational level, and were more likely to be unmarried than the home working group.

**Conclusion:**

Our findings support the notion of the negative impact of the COVID-19 pandemic on mental health in both office workers and those working from home. However, the group who worked from home seem to be more resilient with fewer psychiatric symptoms and better functioning.

## Introduction

In December 2019, the coronavirus disease 2019 (COVID-19) was first recognized as a disease caused by the severe acute respiratory syndrome coronavirus (SARS-CoV-2) with its initial infection site in Wuhan, Hubei Province, China.^1^ Initially, the disease was believed to be confined to this area, but it quickly spread worldwide and there have been 532,201,219 confirmed cases of COVID-19 and 6,305,358 people have died around the world due to COVID-19 (as of 10th June 2022).^2,3^ The COVID-19 pandemic has had a negative impact on everyday life, has threatened people’s health both mentally and physically, and has impaired social and economic development.^4-6^

The burden of COVID-19 cases forced many countries around the world to impose lockdowns and social distancing practices, to avoid the spread of the coronavirus until vaccines arrived. However, many services were not interrupted, and a considerable number of employees working onsite faced a high risk of infection by the new coronavirus, including custodial staff and orderlies in hospitals, as well as teachers and child-care workers, grocery clerks and supermarket workers, delivery people, factory and farm workers, and restaurant staff.^7^ Furthermore, health care workers are in direct contact with coronavirus-infected patients in hospitals and are thus at increased risk of SARS-CoV-2 infection.^5-8^

Experience from previous pandemics showed that some factors might affect mental health in the general population, such as quarantine, fear of infection, frustration and boredom, inadequate supplies, and a lack of information.^6,7^ However, employees working onsite might face additional challenges in the workplace, including lack of adequate distancing, lack of personal protection equipment, work overload, and deaths related to COVID-19, often compounded by the need to commute to work by public transport.^9-11^ In addition, early career and young health care workers and women are more vulnerable to additional impacts of mental health in the workplace.^11,12^ Taken together, these factors suggest employees working in the office are at a higher risk of psychological distress than those working from home, who do not face direct contact with sources of infection during the COVID-19 pandemic.

A meta-analysis of cross-sectional studies reported relatively high rates of anxiety, depression, post-traumatic stress disorder (PTSD), psychological distress, and stress in the general population during the COVID-19 pandemic in eight countries^13^ and similar data were reported in Brazil.^14^ In particular, studies focused on working in the office also showed a substantial burden of mental health symptoms in this population in distinct cultures. In China, a cross-sectional study showed a considerable proportion of symptoms of depression, anxiety, insomnia, and distress in health care workers, with more severe symptoms in workers on the front line.^10^ Italy was one of the most severely affected countries in terms of the number of deaths in the ongoing pandemic and health care workers in the country reported high levels of depression, anxiety, and post-traumatic stress symptoms, with an increased risk for PTSD among front line workers.^15^ In the United States, Young et al.^16^ reported that approximately 40% of medical staff suffered from mood disorders during the pandemic. In Canada, the prevalence of anxiety and depression among medical staff was also significant.^17^ A study conducted by Fournier et al.^11^ demonstrated that the pandemic had a marked psychological impact on all professionals working in health care facilities in France, mainly due to increased stress related to the pandemic.

Thus far, no studies have compared mental health outcomes, functioning, and quality of life (QoL) between individuals working from home and those working in the office in a sample selected during the COVID-19 pandemic. Our hypothesis is that employees who worked from home experienced lower levels of distress, anxiety, depression and, consequently, better functioning and QoL than those who worked in the office. Therefore, this study aimed to compare mental health outcomes, functioning, and QoL between individuals who worked from home and those who worked in the office.

## Methods

### Study population

We administered a cross-sectional web-based survey using an anonymous online questionnaire available via social networks (Facebook, Instagram, and Twitter), using a convenience sampling strategy to target members of the adult Brazilian population. Data were collected between May 20th and September 13th, 2020, the first peak period of COVID-19 contagion in Brazil. Individuals working from home were identified by the question “Do you work from home?” The online questionnaire consisted of sociodemographic items, questions used to assess participants’ knowledge regarding COVID-19, prevalence of previous psychiatric disorders and previous chronic disease, symptoms of COVID-19, attitudes and practices with respect to COVID-19, QoL, cognitive functioning, the severity of depression and anxiety, and symptoms of PTSD. Approval for this study was obtained from the local institutional review board at Hospital de Clínicas de Porto Alegre (CAAE 30741920.8.0000.5327). Online informed consent was obtained from the participants.

### Measures

#### Knowledge regarding COVID-19

The knowledge portion of the questionnaire consisted of 10 questions regarding clinical characteristics and prevention of COVID-19. These questions were answered on a true/false basis with an additional “I don’t know” option.^18^ The proportions of correct answers were analysed.

#### QoL

QoL was assessed with the European Health Interview Surveys QoL instrument (EUROHIS-QOL) consisting of eight items (overall QoL, general health, energy, activities of daily living, self-esteem, social relationships, finances, and home). Each item is rated on a five-point response scale^19^ based on the 2 weeks prior to survey participation. The total score is the sum of each item, with higher scores indicating better QoL.

#### Psychosocial functioning

The Functioning Assessment Short Test (FAST)^20^ was used to assess multiple areas of functioning, namely, autonomy, work, cognition, finance, interpersonal relationships, and leisure. For the purposes of the present study, we used the FAST online scale to allow for assessment of the degree of functional impairment experienced during the COVID-19 pandemic. Items in each domain were rated on a four-point scale based on the 2 weeks prior to survey participation. The total score is the sum of each item, and a higher score indicates poor functioning. The FAST online was validated in a sample from the Brazilian population during the COVID-19 pandemic, presenting satisfactory psychometric properties.^21^

#### Psychiatric assessment

The severity of depression, anxiety, and stress was measured as follows:

- The Patient-Reported Outcomes Measurement Information System (PROMIS) for depression (PROMIS Short Form v1.0 - Depression 8a) assesses negative mood (sadness, guilt), views of self (self-criticism, worthlessness), and social cognition (loneliness, interpersonal alienation), as well as decreased positive affect and engagement (loss of interest, meaning, and purpose).^22,23^
- The PROMIS Anxiety assesses self-reported fear (fearfulness, panic), anxious misery (worry, dread), hyperarousal (tension, nervousness, restlessness), and somatic symptoms related to arousal (racing heart, dizziness).^22,23^
- The Impact of Event Scale-Revised (IES-R) is a self-rated, 22-item questionnaire divided into three domains (avoidance, intrusion, and hyperarousal), which evaluates the distress caused by a traumatic event. Each item is rated on a 5-point response scale (0 = not at all; 1 = a little bit; 2 = moderately; 3 = quite a bit; 4 = extremely). The IES-R total score is the sum of the average of each domain. A total score greater than 5.6 indicates psychological stress.

Each of the PROMIS instruments used comprises an eight-item questionnaire that assesses symptoms over the previous 7 days, with each item rated on a 5-point response scale (1 = never; 2 = rarely; 3 = sometimes; 4 = often; 5 =always). All PROMIS scores are presented as T-scores calculated by the Health Measures Scoring Service (https://www.assessmentcenter.net/ac_scoringservice) from the raw sum score, using T-scores from the United States general population. A T-score is a standardized score, with a mean of 50 and a standard deviation (SD) of 10. For depression and anxiety, a T-score lower than or equal to 55 indicates no significant symptoms, a higher score, from 55 to 60 indicates mild symptoms, a score from 60 to 70 indicates moderate symptoms, and a score from 70 to 84.1 indicates severe symptoms. For the purpose of our study, we classified both PROMIS Depression and PROMIS Anxiety T-scores into two categories of severity: no significant/mild symptoms (normal/mild symptoms) and moderate/severe symptoms.^24,25^

## Statistical analysis

Descriptive statistics (number and %) were used to present sociodemographic and clinical characteristics. We used Mann-Whitney or chi-square tests for comparisons between groups as appropriate. We used linear regression to identify potential variables associated with mental health outcomes (e.g., stress, anxiety, and depression) and the potential confounders gender (male as reference category), age, marital status (married or in a stable relationship as reference category), household income (lower income as reference category), days of social distancing, previous psychiatric disorders (free from psychiatric disorders as reference category), and educational level (lower educational level as reference category) for working in the office and working from home. Analyses were performed with SPSS version 18. Statistical significance was set at p < 0.05, and all tests were two-tailed.

## Results

### Sample characteristics

A total of 4,069 participants read and agreed to answer the survey and 3,023 individuals completed the survey. Of these 3,023 respondents, 1,685 reported working in the office and 1,338 were working from home. Those working in the office were younger (years) (31 vs. 33, *U* = 1,006,461, p < 0.001) and more likely to be female (87.4 vs. 81.2%, p < 0.001) than those working from home. A greater percentage of those working in the office were in the lower household income level (46.8 vs. 21.3%, p < 0.001), had a low educational level (57 vs. 25.4%, p < 0.001), and were unmarried (57.9 vs. 53.5%, p < 0.001) compared to those working from home (Table 1). Other sample characteristics are reported in Table 1.

**Table 1:** Characteristics and mental health symptoms in people working from home and working in the office (n = 3,023)

Variables	Work from home (n = 1,338)	Work in the office (n = 1,685)	Statistic	df	p-value
Age, Mdn (Q1,Q3)	33 (25,43)	31 (23,41)	U = 1006461	-	< 0.001
Household, Mdn (Q1,Q3)	3 (2,4)	3 (2,4)	U = 981404	-	< 0.001
Social distancing*, Mdn (Q1, Q3), days	85 (64, 120)	88 (60, 103.5)	U = 959606	-	< 0.01
Household income (BRL),^†^ n (%)					
> 10,386.52	350 (26.2)	204 (12.1)	χ^2^ = 238.39	2	< 0.001
> 2,965.69 to 10,386.52	703 (52.5)	692 (41.1)			
< 708.19 to 2,965.69	285 (21.3)	789 (46.8)			
Gender, n (%)					
Women	1,082 (81.2)	1,461 (87.4)	χ^2^ = 21.98	1	< 0.001
Men	251 (18.8)	211 (12.6)			
Marital status, n (%)					
Married	622 (46.5)	709 (42.1)	χ^2^ = 5.89	1	< 0.001
Unmarried	716 (53.5)	976 (57.9)			
Education, n (%)					
Graduated	998 (74.6)	725 (43.0)	χ^2^ = 303.1	1	< 0.001
Ungraduated	340 (25.4)	960 (57.0)			
PROMIS Depression, n (%)					
Normal/mild	521 (38.9)	473 (28.1)	χ^2^ = 39.91	1	< 0.001
Moderate/severe	817 (61.1)	1,212 (71.9)			
PROMIS Anxiety, n (%)					
Normal/mild	247 (18.5)	231 (13.7)	χ^2^ = 12.65	1	0.111
Moderate/severe	1,091 (81.5)	1,454 (86.3)			
FAST COVID-19, Mdn (Q1,Q3), score	23 (15, 32)	27 (17, 38)	U = 940188.5	-	< 0.001
EUROHIS-QOL, Mdn (Q1,Q3), score	27 (22, 31)	24 (20, 28)	U = 876124	-	< 0.001
IES-R, n (%)					
Symptoms of PTSD	353 (26.4)	656 (38.9)	χ^2^ = 52.82	1	< 0.001

df = degrees of freedom; Mdn = median; PROMIS = Patient-Reported Outcomes Measurement Information System; PTSD = post-traumatic stress disorder; EUROHIS-QOL = European Health Interview Surveys QoL instrument; IES-R = Impact of Event Scale-Revised.

* Work from home (n = 1,316), work in the office (n = 1,557); ^†^ 1 BRL = 0.20 USD.

### Psychological impact, depression and anxiety symptoms

The psychological impact of COVID-19, as assessed by the IES-R scale, revealed that those who worked from home had a lower prevalence (61.1 vs. 73.6%, p < 0.001) of symptoms of post-traumatic stress than those who worked in the office. Depression assessed by PROMIS Depression showed that those working from home had a lower prevalence of moderate to severe depression symptoms than those working in the office (71.9 vs. 61.1%, p < 0.001). However, the prevalence of moderate to severe anxiety assessed by PROMIS Anxiety was similar in the two groups (86.3 vs. 81.5%, p = 0.11) (Table 1).

### Previous psychiatric disorders

The prevalence of self-reported psychiatric disorders was greater in people working in the office than in people working from home (43.4 vs. 38.8%, respectively, p = 0.01). Those working from home had lower prevalence of depression (23.4 vs. 29.6%, p <0.001), self-reported panic disorders (6.1 vs. 10.7%, p <0.001), and social phobia (2.1 vs. 4.5% p < 0.001) than those working in the office (Table 2).

**Table 2:** Self-reported psychiatric disorders in people working from home and working in the office (n = 3,023)

Variables	Work from home (n = 1,338)	Work in the office (n = 1,685)	χ^2^	df	p-value
Any psychiatric disorder	519 (38.8)	732 (43.4)	6.656	1	0.010
Main diagnosis					
Depression	313 (23.4)	498 (29.6)	14.424	1	< 0.001
Panic disorder	81 (6.1)	181 (10.7)	20.707	1	< 0.001
Generalized anxiety disorder	291 (21.7)	430 (25.5)	5.84	1	0.016
Obsessive-compulsive disorder	27 (2.0)	51 (3.0)	3.019	1	0.082
Social phobia	28 (2.1)	75 (4.5)	12.60	1	< 0.001
Bipolar disorder	46 (3.4)	88 (5.2)	5.607	1	0.018
Schizophrenia	1 (0.1)	4 (0.2)	1.195	1	0.274
Drug abuse	11 (0.8)	17 (1.0)	0.284	1	0.594
Post-traumatic stress disorder	37 (2.8)	67 (4.0)	3.292	1	0.070
Other diagnoses	58 (4.3)	71 (4.2)	0.027	1	0.870

Data presented as n (%).

df = degrees of freedom.

### Knowledge about COVID-19

Regarding knowledge about COVID-19, those working in the office and working from home had similar responses, except for one question: “Persons with COVID-2019 cannot infect others when a fever is not present” (93.9 vs. 87.4%, p <0.001) (Table 3). During the ongoing pandemic, people working in the office had higher prevalence of COVID-19 diagnoses (4 vs. 2.2%, p < 0.01), need to visit a doctor (10.3 vs. 5.1%, p < 0.01), having met someone with COVID-19 (24.5 vs. 14.1%, p < 0.001), and loss of a loved one to COVID-19 (8.4 vs. 5.6%, p < 0.01) (Table 4).

**Table 3:** Correct answers to questions about knowledge about COVID-19 of people working from home and working in the office (n = 3,023)

Answers	Work from home (n = 1,338)	Work in the office (n = 1,685)	χ^2^	df	p-value
1. The main clinical symptoms of COVID-19 are fever, fatigue, dry cough, and myalgia. (True)	1,147 (85.7)	1,401 (83.1)	3.75	1	0.053
2. There currently is no effective cure for COVID-2019, but early symptomatic and supportive treatment can help most patients recover from the infection. (True)	1,242 (92.8)	1,555 (92.3)	0.315	1	0.575
3. Not all persons with COVID-2019 will develop severe cases. Only those who are elderly, have chronic illnesses, and are obese are more likely to be severe cases. (True)	810 (60.5)	1,048 (62.2)	0.865	1	0.352
4. Eating or coming into contact with wild animals would result in infection by the COVID-19 virus. (False)	975 (72.9)	1,187 (70.4)	2.153	1	0.142
5. Persons with COVID-2019 cannot infect others when a fever is not present. (False)	1,256 (93.9)	1,472 (87.4)	35.92	1	< 0.001
6. The coronavirus spreads via respiratory droplets of infected individuals. (True)	1,312 (98.1)	1,639 (97.3)	1.99	1	0.159
7. It is not necessary for children and young adults to take measures to prevent the infection by the coronavirus. (False)	1,314 (98.2)	1,656 (98.3)	0.023	1	0.880
8. To prevent infection by COVID-19, individuals should avoid going to crowded places such as train stations and avoid taking public transportations. (True)	1,297 (96.9)	1,629 (96.7)	0.161	1	0.688
9. Isolation and treatment of people who are infected with the COVID-19 virus are effective ways to reduce the spread of the virus. (True)	1,303 (97.4)	1,634 (97)	0.455	1	0.500
10. People who have contact with someone infected with the COVID-19 virus should be immediately isolated in a proper place. In general, the observation period is 14 days. (True)	1,303 (97.4)	1,622 (96.3)	2.999	1	0.083

Data presented as n (%).

COVID-19 = coronavirus disease 2019; df = degrees of freedom.

**Table 4:** Events, attitudes, and practices related to COVID-19 among people working from home and working in the office (n = 3,023)

Events, attitudes, and practices	Work from home (n = 1,338)	Work in the office (n = 1,685)	χ^2^	df	p-value
Visited a doctor	68 (5.1)	173 (10.3)	27.33	1	< 0.01
Positive COVID-19 diagnosis	29 (2.2)	67 (4.0)	7.94	1	< 0.01
Needed hospitalization	1 (0.1)	7 (0.4)	3.28	1	0.70
Met someone with COVID-19	189 (14.1)	413 (24.5)	50.43	1	< 0.001
Lost a loved one to COVID-19	75 (5.6)	141 (8.4)	8.58	1	< 0.01
Positive attitudes					
Agree that COVID-19 will finally be successfully controlled	366 (27.4)	510 (30.3)	3.77	2	0.152
Confident that Brazil can win the battle against the coronavirus	845 (63.2)	1,150 (68.2)	8.63	1	< 0.01
Positive practices					
Has not gone to crowded places in recent days	131 (9.8)	257 (15.3)	19.88	1	<0.001
Wore a mask when leaving home in recent days	1,325 (99.0)	1,663 (98.7)	0.73	1	0.39

Data presented as n (%).

COVID-19 = coronavirus disease 2019; df = degrees of freedom.

### Functioning and QoL

Those working from home had lower scores on the FAST scale (24 vs. 28, t = 8.58, p < 0.001), suggesting greater functionality, and higher scores on the EUROHIS-QOL questionnaire (26 vs. 24, t = -10.67, p < 0.001), indicating better QoL, than those working in the office (Table 1).

### Multivariate analysis

We used linear regression models to analyse determinant factors associated with the scores on IES-R, depressive PROMIS Depression scores, and anxious PROMIS Anxiety scores. In addition, we also included work status (working in the office or working from home) during the pandemic in the models. All models were statistically significant for predicting higher or lower IES scores (F = 50.921, degrees of freedom [df] = 10, p < 0.001) and presence of depression (F = 104.195, df = 10, p < 0.001) and anxiety (F = 91.160, df = 10, p < 0.001). In addition, the final models explained 15.2% of the variance in IES scores, 23.5% of the variance in depression scores, and 21.2% of the variance in anxiety scores.

For the IES scores, seven variables significantly contributed to the model: gender (B = 1.9, 95% confidence interval [95%CI] 0.93 to 1.46, p < 0.001), age (B = -0.03, 95%CI -0.04 to -0.02, p < 0.001), educational level (B = -0.37, 95%CI -0.58 to -0.16, p < 0.01), household income level (medium income B = -0.91, 95%CI -1.13 to -0.69, p < 0.001; high income B = -1.45, 95%CI -1.74 to -1.16, p < 0.01), history of psychiatric illness (B = 0.88, 95%CI 0.69 to 1.07, p < 0.001), and contact with someone with COVID-19 (B = 0.36, 95%CI 0.12 to 0.61, p < 0.01). Therefore, female gender, younger age, lower educational level, lower income, a self-reported history of psychiatric illness, and contact with someone with COVID-19 were associated with higher IES-R scores (Table 5).

**Table 5:** Associations between PTSD, depression, and anxiety symptoms and gender, age, educational level, household income level, history of psychiatric illness, contact with someone with COVID, and marital status

Variable	IES-R			PROMIS Depression			PROMIS Anxiety		
	B	95%CI	p-value	B	95%CI	p-value	B	95%CI	p-value
Intercept	4.76	4.28 to 5.24	< 0.001	61.85	60.48 to 63.21	< 0.001	66.74	65.41 to 68.14	< 0.001
Gender	1.19	0.93 to 1.46	< 0.001	3.41	2.64 to 4.17	< 0.001	3.86	3.09 to 4.63	< 0.001
Age	-0.03	-0.04 to -0.02	< 0.001	-0.19	-0.21 to -0.17	< 0.001	-0.17	-0.19 to -0.15	< 0.001
Educational level	-0.37	-0.58 to 0.16	< 0.001	-1.08	-1.67 to 0.48	< 0.01	-1.12	-1.72 to 0.52	< 0.001
Household income level									
Medium	-0.91	-1.13 to -0.69	< 0.001	-2.56	-3.19 to -1.94	< 0.001	-2.17	-2.80 to -1.54	< 0.001
High	-1.45	-1.74 to -1.16	< 0.001	-4.56	-5.39 to -3.72	< 0.001	-4.14	-4.98 to -3.30	< 0.01
History of psychiatric illness	0.88	0.69 to 1.07	< 0.001	4.20	3.66 to 4.75	< 0.001	4.14	3.60 to 4.70	< 0.001
Contact with someone with COVID-19	0.36	0.12 to 0.61	< 0.01	0.327	0.371 to 1.026	0.358	1.05	0.34 to 1.75	< 0.01
Marital status	0.031	-0.171 to 0.233	0.763	1.17	0.58 to -1.75	< 0.001	0.429	-0.154 to 1.011	0.149

95%CI = 95% confidence interval; COVID = coronavirus disease; IES-R = Impact of Event Scale-Revised; PROMIS = Patient-Reported Outcomes Measurement Information System; PTSD = post-traumatic stress disorder.

For depression scores, seven variables significantly contributed to the model: gender (B = 3.41, 95%CI 2.64 to 4.17, p < 0.001), age (B = -0.19, 95%CI -0.21 to -0.17, p < 0.001), educational level (B = -1.08, 95%CI -1.67 to -0.48, p < 0.01), household income (medium income B = -2.56, 95%CI -3.19 to -1.94, p < 0.001; high income B = -4.56, 95%CI -5.39 to -3.72), history of psychiatric illness (B = 4.20, 95%CI 3.66 to 4.75, p < 0.001), and marital status (B = 1.17, 95%CI 0.58 to 1.75, p < 0.001). Therefore, female gender, younger age, lower educational level, lower income, self-reported history of psychiatric illness, and being single were associated with higher PROMIS Depression scores (Table 5).

For anxiety scores, seven variables significantly contributed to the model: gender (B = 3.86, 95%CI 3.09 to 4.63, p < 0.001), age (B = -0.17, 95%CI -0.19 to -0.15, p < 0.001), educational level (B = -1.12, 95%CI -1.72 to -0.52, p < 0.001), household income (medium income B = -2.17, 95%CI -2.80 to -1.54, p < 0.001; high income B = -4.14, 95%CI -4.98 to -3.30, p < 0.01), history of psychiatric illness (B = 4.14, 95%CI 3.60 to 4.70, p < 0.001), and contact with someone with COVID-19 (B = 1.05, 95%CI 0.34 to 1.75, p < 0.01). Therefore, female gender, younger age, lower educational level, lower income, self-reported history of psychiatric illness, and contact with someone with COVID-19 were associated with higher PROMIS Anxiety scores (Table 5).

## Discussion

The present study compared mental health outcomes, functioning, and QoL and associated variables between people working in the office and working from home during the first wave of the ongoing COVID-19 pandemic in Brazil. We show that both groups experienced a negative impact from COVID-19 on their mental health during the ongoing pandemic; however, participants who worked from home experienced lower levels of anxiety, stress, and depression than participants who worked in the office, even after controlling for possible confounders. Regarding sociodemographic characteristics, people working in the office were younger, female, had lower family income, were more likely to be single, and had lower levels of education. These factors may have contributed to the harmful effects of the COVID-19 pandemic, according to recent studies.^16-26^

In addition, the psychological distress experienced by both groups (those working in the office and those working from home) corroborates other recent studies^5,10,11,26-30^ and, to some extent, can be explained by psychological overload, problems sleeping, physical distancing, and fear of spreading the virus.^5,11,20,26,27,29^ The prevalence of mental health problems in our sample, in both those working in the office and those working from home, somewhat agreed with studies performed in other countries.^13,15,26,31-33^ In the working in the office subset, our results for the rates of symptoms of depression (71.9%) and anxiety (86.3%) were higher than those in a meta-analysis of cross-sectional studies in the general population, which reported prevalence of symptoms of depression as high as 48.3% and of anxiety as high as 50.6%.^13^ However, post-traumatic stress symptoms (38.9%) were in line with the range found in other countries (from 7 to 53.8%) in the same meta-analysis.^13^

Comparing people working in the office with people working from home, we found more symptoms of depression and distress in the former. Our study is similar to research conducted in a Spanish population that found mental health problems in health professionals^31^ and particularly in those individuals that were working on the frontline.^34^ In Italy, the prevalence of PTSD symptoms in front line health care workers was around 50% and slightly higher than the prevalence found in our in-office workers.^15^ In China, a cross-sectional study in a single centre reported a smaller proportion of participants with severe symptoms of depression and anxiety among medical and administrative staff compared to the rate in our subset of people working in the office.^32^ Finally, our data diverge from those of a cross-sectional study in the United Kingdom which showed that respondents self-declared as working in the office had lower levels of depressive symptoms than those working from home.^33^ In summary, some of the differences between studies are probably because we assessed people of any profession working in the office, in contrast with previous studies that reported results for health care workers only, as well as sociocultural differences. According to Sole et al.,^35^ psychiatric symptoms have traditionally been associated with poor functioning in clinical samples, in agreement with our study that lower psychiatric symptoms were associated with better functioning.

Our study showed that people who worked from home had fewer depressive symptoms, stress and anxiety than people who worked in the office, suggesting they were more resilient to cope with the adversities of the pandemic. This hypothesis is supported by previous studies that have shown associations between greater resilience with less severity of depression, suicidal ideation, and anxiety symptoms, and less concern about the effects of COVID-19.^36,37^ Additional studies are needed to assess mental health outcomes in the ongoing pandemic considering resilience as a mediator of pandemic stressors regarding working from home.

To the best of our knowledge, this is the first study to report data comparing mental health status between Brazilians working in the office and working from home during the ongoing COVID-19 pandemic. Interpretation of our results should consider some limitations of the study. First, we used an online survey with a convenience sample method, which may not yield a representative sample of the overall Brazilian office worker and home worker population. Second, the subset working in the office was not drawn only from health care workers, but also included essential non-health care workers, which might constitute a bias, since the former were more exposed to infection by SARS-CoV-2 and death from COVID-19 than the latter. Third, all outcomes were self-reported instead of evaluated by a clinician. Finally, there is a chance that only individuals who were struggling with their mental health during the pandemic would be interested in answering the questionnaire.

## Conclusion

Our findings support the notion of the negative impact of the COVID-19 pandemic on mental health among both people working in the office and people working from home. However, in our study, those working from home experienced lower levels of stress, anxiety, and depression than those working in the office, even after controlling for potential confounders. These findings suggest that working from home may reduce the negative effects of the ongoing pandemic in terms of symptoms of depression and PTSD, most likely because of greater resilience and knowledge about COVID-19.
